# A feasibility hybrid II randomised controlled trial of volunteer ‘Health Champions’ supporting people with serious mental illness manage their physical health: study protocol

**DOI:** 10.1186/s40814-021-00854-8

**Published:** 2021-05-31

**Authors:** Julie Williams, Elliann Fairbairn, Ray McGrath, Ioannis Bakolis, Andy Healey, Ubong Akpan, Isabel Mdudu, Fiona Gaughran, Euan Sadler, Zarnie Khadjesari, Kate Lillywhite, Nick Sevdalis

**Affiliations:** 1 Centre for Implementation Science, Health Service and Population Research Department, Institute of Psychiatry, Psychology and Neuroscience, King’s College London, London, UK; 2 South London and Maudsley NHS Foundation Trust, London, UK; 3 King’s Health Partners Mind and Body Programme, Guy’s and St Thomas’ Hospital, Ground Floor, Counting House, Thomas St, London, UK; 4 Department of Biostatistics and Health Informatics, Institute of Psychiatry, Psychology and Neuroscience, King’s College London, London, UK; 5 King’s Health Economics, Health Service and Population Research Department, Institute of Psychiatry, Psychology and Neuroscience, King’s College London, London, UK; 6 Psychosis Studies, Institute of Psychiatry, Psychology and Neuroscience, King’s College London, London, UK; 7 National Psychosis Service, South London and Maudsley NHS Foundation Trust, London, UK; 8 Applied Health Research and Care (ARC), South London, UK; 9 Department of Nursing, Midwifery and Health, School of Health Sciences, Faculty of Environmental and Life Sciences, University of Southampton, Southampton, UK; 10 Behavioural and Implementation Science (BIS) Research Group, School of Health Sciences, University of East Anglia, Norwich Research Park, Norwich, UK

**Keywords:** Serious mental illness, Physical health, Intervention, Volunteers

## Abstract

**Background:**

People with serious mental illnesses (SMI) such as schizophrenia often also have physical health illnesses and interventions are needed to address the resultant multimorbidity and reduced life expectancy. Research has shown that volunteers can support people with SMI. This protocol describes a feasibility randomised controlled trial (RCT) of a novel intervention involving volunteer ‘Health Champions’ supporting people with SMI to manage and improve their physical health.

**Methods:**

This is a feasibility hybrid II randomised effectiveness-implementation controlled trial. The intervention involves training volunteers to be ‘Health Champions’ to support individual people with SMI using mental health services. This face-to-face or remote support will take place weekly and last for up to 9 months following initial introduction. This study will recruit 120 participants to compare Health Champions to treatment as usual for people with SMI using secondary community mental health services in South London, UK. We will measure the clinical and cost effectiveness including quality of life. We will measure the implementation outcomes of acceptability, feasibility, appropriateness, fidelity, barriers and enablers, unintended consequences, adoption and sustainability.

**Discussion:**

There is a need for interventions to support people with SMI with their physical health. If this feasibility trial is successful, a definitive trial will follow to fully evaluate the clinical, cost and implementation effectiveness of Health Champions supporting people with SMI.

**Trial registration:**

ClinicalTrials.gov, registration no: NCT04124744.

## Background

People diagnosed with a serious mental illness (SMI) such as schizophrenia, bipolar disorder and major depression have poorer physical health and experience more multimorbidity than the general population [1–3]. People with SMI have higher rates of cardiovascular disease [4], diabetes [5] and respiratory disease [6]. Recent research has shown that this health gap is widening [2].

The reasons for this inequality are complex, encompassing individual, health service and societal factors [7]. Individual level factors include severity of illness and symptoms which can affect how people access help. The health service factors include fragmentation of physical and mental health services, the impact of cuts in services, and ‘diagnostic overshadowing’ where physical health symptoms are seen as part of mental illness by health professionals. Social factors include stigma, poverty, poor housing [7] and social isolation [8]. There is a clear need for interventions that promote the physical health of this population at all of these levels [9]. Solutions need to be flexible enough to meet the needs of individual service users and this can be difficult in England within over-stretched primary care and mental health services [10].

There is little evidence on how best to support people with SMI to manage their physical health. A Cochrane review of physical health advice for service users with SMI demonstrated a modest improvement in quality of life [11]. However, the review also highlighted a significant research gap in assessing physical health advice interventions and their impact on service users’ physical health. This research gap was also found in a recent systematic review of physical health screening in this population [12]. A recently published Lancet Psychiatry Commission summarises the best available evidence for interventions targeting specific health outcomes such as smoking and obesity and advocates community-based interventions [13].

In this protocol, we describe a novel intervention in which volunteers will work alongside people using community mental health services for up to 9 months to support them with managing their physical health. There is emerging literature on the benefits of mental health service users being supported by volunteers both for the service user and volunteer. For service users, this includes contact with someone non-judgemental who helps facilitate increased social interaction, and for volunteers, the feeling they are giving something back and helping others [14–16]. However, there is little evidence regarding volunteers working with service users specifically on their physical health. This study aims to contribute to this much needed area by evaluating the introduction of volunteer ‘Health Champions’ who are matched with service users to support them in the community in setting and working towards their own defined physical health goals as part of a randomised controlled trial.

The aims of this study are to evaluate the feasibility of deploying trained volunteer ‘Health Champions’ to support people with SMI in managing their physical health, compared to treatment as usual. We aim to collect clinical, economic and implementation measures to assess the feasibility and to understand the implementation challenges of undertaking the intervention in routine practice.

As the study will start during the COVID-19 pandemic, contacts will be face-to-face or remote, in line with contemporaneous national and local guidance and approvals.

### Development of the intervention

#### Phase 1 (February 2018-January 2019): Initial conceptualisation

The intervention was developed as part of a larger ‘Integrated Mental and Physical Health Systems’ (IMPHS) Programme funded by the Maudsley Charity and involving SLaM service users and providers. The initial conceptualisation of the intervention came from discussion between the Maudsley Charity, the SLaM Volunteer Manager and the IMPHS project scientists. SLaM volunteers already support people in a variety of ways including befriending and the scope to use volunteers to support physical health was developed into an idea for an intervention. Advice was sought from the King’s Health Partners (KHP) Mind and Body Programme Expert Advisory Group and the SLaM Serious Mental Illness Service User Group as part of the initial development of the Health Champions project.

#### Phase 2 (February-October 2019): Development of a theory of change

Initial conceptualisation of the intervention was followed by development of a Theory of Change (ToC) for the study. A draft ToC was produced by the IMPHS project team, which was subsequently reviewed and refined in two stakeholder workshops (on 30 September and 1 October 2019) with 22 participants. ToC workshop participants were service users, carers, clinical staff, voluntary sector representatives and volunteers.

The ToC that emerged from this process (see Table 1) proposes that, by working with a Health Champion, participants will improve their own self-management of their physical health by goal-setting and problem-solving with their Health Champion. This may lead to improvement in their physical and mental health and a decrease in their treatment burden. All of these will then lead to improved physical and mental quality of life. The ToC also proposes a possible decrease in loneliness both for the duration of the intervention and afterwards.

**Table 1:** Theory of change-modified items from Stakeholder Workshops in bold

Inputs/resources	Facilitators/barriers	Actions/activities	Outcomes	Impacts
Project staff SLaM volunteer manager Health Champions Volunteer Coordinator **Service user and carer steering group** Volunteers SLaM staff and infrastructure **Participants** **Carers** Local community organisations e.g.: tba Training and supervision for health champions **Small amount of funding for activities during the intervention**	Facilitators: Large number of existing volunteers within SLaM Knowledge and experience of project team Support from SLaM **Skills and experience of Health Champions** Barriers: Ability to recruit enough health champions New role—needs to be clearly explained **Poverty and housing issues for participants**	Recruit Health Champions **Recruit participants and ensure they have full details of the intervention** **Training of Health Champions including on how to build relationships and coaching skills** **Support and supervision for Health Champions during the intervention** **Effective matching of Health Champions and participants** Mapping of voluntary and community sector organisations Role of Health Champions **Help to set goals** • To give light-touch education and information to patients to promote healthy lifestyles Specific activities for patient-Health Champion pairs to do together may include: • Encouraging participation and engagement in healthy hobbies and activities in the community—for instance, swimming, walking, use of gyms/exercise facilities (e.g. park gyms, and local authority sports centres) • Taking part in group activities and exercise—building social networks, self-esteem and confidence • Providing tips and guidance around nutrition, healthy cooking and eating (including food shopping and food traffic light systems), and cooking together • Helping patients attend follow up GP or outpatient appointments for physical health issues **Groups for Health Champions only and Health Champions and participants to meet and discuss intervention/progress/share ideas** **Possible teaching sessions for Health Champions and participants on areas of interest**	For participants Increased confidence in dealing with physical health including ability to attend physical health appointments Increased knowledge of how to deal with physical health concerns and problems Increase in doing things to improve physical health-e.g. being more physically active, change in diet **Increase in enjoyable activities** Reduced levels of social isolation Increase in level of health screenings **Improved mental health** **For Health Champions** **Improved health** **Learning new skills** **Ability to give back** **For SLaM services** **Support with the participants they work with** **Better knowledge of local community organisations**	Improved quality of life Improved physical health of patients Improved patient experience and engagement Improved links with the voluntary and community sector to address physical and mental health Increased awareness and understanding amongst volunteers, patients and carers of physical health and its interaction with mental health, and how to promote healthy lifestyles Increased awareness and understanding of the types of services (and who would most benefit) provided by the voluntary and community sector

#### Phase 3 (March 2019-December 2019): Operationalisation of the intervention

Whilst the ToC was being developed and consulted on, we finessed and operationalised the intervention. This included developing the processes for recruiting and training the volunteers to deliver the intervention (see Section 2.7.1 for details), and for recruiting people with SMI as participants into the trial (see Section 2.8).

### Feasibility outcomes

We will measure the following feasibility outcomes:

1. Feasibility of the intervention measured using the Feasibility of Intervention Measure [17] and qualitative interviews with participants and Health Champions.
2. Appropriateness of the intervention to both participants and Health Champions measured using the Intervention Appropriateness Measure [17] and qualitative interviews with participants and Health Champions.
3. Fidelity to the intervention which we will measure using a content analysis of Health Champions and participants journals to assess fidelity of the delivery of the intervention and fidelity of receipt of the intervention, supervision records, and the number and percentage of meetings between each Health Champion and participant.
4. Barriers and facilitators and unintended consequences will be measured using qualitative interviews with a subset of 30% participants and Health Champions chosen at random after the intervention recording whether the intervention was delivered face to face or remotely plus details from the journals and supervision records. We will also interview all participants who drop out of the intervention to understand their reasons for this.
5. Adoption of the intervention will be measured by the number and percentage of participants that start the intervention, how many sessions they have and how long (in months) they stay in the intervention.
6. Adoption and sustainability will also be assessed using interviews with SLaM managers and commissioners.

## Methods and analysis

### Design

This is a feasibility hybrid II effectiveness-implementation randomised controlled trial. Hybrid type II trials [18] are at the cutting edge of applied health research and are used to jointly assess the clinical effectiveness and implementation strategies of evidence-based interventions so that findings can be interpreted in the light of how best to implement such interventions at scale and with sustainability, including economic viability. The schedule of enrolment, interventions and assessments is shown in Table 2.

**Table 2:** Data collection plan

	Baseline assessment	Follow-up assessment 1 (at end of intervention or after 9 months)	Follow-up assessment 2 (6 months after end of intervention or 9 month assessment)
**Participants**			
Patient information and informed consent	X		
Demographics	X		
EQ-5D-5L	X	X	X
ReQOL	X	X	X
Patient activation measure	X	X	X
Multimorbidity Treatment Burden Questionnaire	X	X	X
De Jong Gierveld Loneliness Scale	X	X	X
Participant goals	X	X	X
Health Questionnaire including number of health screenings and service use in last 6 months	X	X	X
Acceptability 1. Acceptability of intervention measure 2. Interview		X	
Appropriateness 1. Intervention appropriateness measure 2. Interview		X	
Feasibility 1. Feasibility of intervention measure 2. Interview		X	
Fidelity-interview		X	
Barriers and facilitators-interview		X	
Adoption-interview		X	X
Sustainability- Interview		X	X
Unintended consequences-interview		X	X
Experience of the intervention-interview		X	
**Health champions**			
Acceptability 1. Acceptability of intervention measure 2. Interview		X	
Appropriateness 1. Intervention appropriateness measure 2. Interview		X	
Feasibility 1. Feasibility of intervention measure 2. Interview		X	
Fidelity-interview		X	
Barriers and facilitators-interview		X	
Unintended consequences-interview		X	
Experience of the intervention-interview		X	

### Study setting

Participants will be recruited from Community Mental Health Teams (CMHTs) in the South London and Maudsley NHS Trust (SLaM: London, UK) working with people with SMI. The intervention will be delivered in the community.

### Participants

Participants will be people using the participating CMHTs.

#### Inclusion criteria

- 18 years and older
- Diagnosed with a SMI including schizophrenia, bipolar disorder, schizoaffective disorder, delusional and other non-mood psychotic disorders and major depression
- Has capacity to give written informed consent to take part in the trial
- Able to provide a named Care Coordinator or other Point of Contact in the CMHT reachable in the event of a health crisis
- Wants to make changes to their physical health (we will ask referrers to have a conversation with anyone being referred to ask this question and this will also be asked by the researcher who first contacts the potential participant)

#### Exclusion criteria

- Under 18 years of age
- Unable to give informed consent

### Ethical approval

Ethical approval for the trial has been obtained from Brent Research Ethics Committee, REC reference no: 20/LO/0214.

### The intervention

#### Overview of the intervention

We will recruit and train volunteers to act as Health Champions. These Health Champions will support individual service users to set and work towards their own individually decided physical health goals. They will meet face-to-face once a week for up to 9 months at a location decided by the participant and in line with their goals or remotely depending on the guidance on social distancing at the time. If the participant reaches their goals within 9 months, they can choose to end their involvement. The Health Champions will be supported by a Volunteer Coordinator who will be based in the SLaM Volunteer team and will be recruited specifically for this role.

### The ‘Health Champions’ intervention

Health Champions will work individually with a matched service user participant over 9 months, meeting weekly for a minimum of 1 h to support the participant to set and work towards their own physical health goal(s). These meetings will be face-to-face depending on the guidance at the current time. We will match the Health Champion and participant based on geographical area and interests. If participants have a preference in terms of being matched in terms of age, gender and ethnicity, we will try to meet this preference.

Once the participant and Health Champion have been matched, there will be an initial meeting with the Volunteer Coordinator present (either face-to-face or remotely) to check if they are happy to work together and answer any questions.

In the initial face-to-face or remote meeting, the participant will be encouraged to set their own physical health goals with their Health Champion. These goals will be logged in a ‘Participant Physical Health Journal’ which is a journal held by the participant where they can record and reflect on their progress. All subsequent meetings between Health Champions and participants should aim to help the participant achieve these physical health goals and log their progress in the ‘Participant Physical Health Journal’. Health Champions will also be asked to complete a ‘Health Champions Journal’ to record their experience as a Health Champion and in supporting the person they are working with. The participant and their Health Champion will be encouraged to complete both journals together during a short reflection period at the end of each weekly session, though they may complete them separately if they wish to. These journals are to help participants and Health Champions record and reflect on the goals set and progress towards them. They also have a secondary purpose of helping to evaluate the fidelity of the intervention (see evaluation section for further details of this). The intervention will take place in the community with the specific location being dependent on the goals of the participants (for example, if they wish to go to the gym etc.) if this is possible. If not, meetings will take place remotely. As this is a feasibility trial, we will record any tailoring or modifications made by any of the Health Champions in response to needs or wishes of their matched participant.

We will also facilitate regular events for Health Champions and participants to meet other Health Champions and participants, to share experiences and resources and provide teaching on particular conditions or activities if a group of participants emerged through the trial with the same goals or health conditions to manage.

### Procedure

#### Health Champion recruitment, training and support

SLaM has a well-established Volunteer Department. For initial Health Champions recruitment, the role of Health Champions will be advertised to the over 300 volunteers at SLaM. Health Champions will be recruited in accordance with SLaM policies including DBS/reference checks and mandatory training for the safeguarding of vulnerable people, lone-working and boundary setting for volunteers. Health Champion eligibility criteria:

- 18 years and over.
- Disclosure and Barring Service (DBS) checked and cleared.
- Able to attend the relevant SLaM Volunteer and Health Champion Programme training and commit 2 h per week for up to 9 months.

To ensure fidelity of the intervention, prior to deployment, Health Champions will receive 1 day remote training on the role of the Health Champion which will include outlining the role of the Health Champion, the importance of physical health and its link to mental health, how to work with people to set their own goals, coaching techniques using the REACH model of coaching [19] and how to build and end an effective relationship with a service user. This training will be provided by the Volunteer Coordinator, research team and experts in coaching. The Health Champions will also receive monthly supervision from the Volunteer Coordinator throughout the study and the opportunity to meet as a group to share experiences and learn from each other. This will ensure that they feel confident to work in the community and develop a good level of knowledge and expertise around supporting physical health in service users with SMIs.

The Volunteer Department in SLaM have an existing process for raising concerns regarding crises, emergencies, safe-guarding and any other issues arising concerning the well-being of volunteers or service users. The Health Champion volunteers will be required to follow these processes and raise any issues with the Volunteer Coordinator and the service user’s Care Coordinator.

The Volunteer Coordinator will be also work with volunteers (not necessarily Health Champions) to identify resources provided by voluntary sector organisations (VSOs) in the London boroughs covered by SLaM (Croydon, Lambeth, Lewisham and Southwark). They will act as a point of contact to negotiate access to resources where there are gaps, foster new relationships and link VSOs. Health Champions will be provided with a database of cost-effective local resources available to service users to help achieve their physical health goals.

### Participant recruitment

We will recruit participants from CMHTs across all four South London boroughs that are covered by SLaM services—namely the boroughs of Lambeth, Lewisham, Southwark and Croydon. Participants will be recruited using two methods.

1. Directly recruited from the community teams by staff identifying and recruiting people who are eligible for the study. Staff will be made aware of the eligibility criteria of the study.
2. Using the SLaM Consent for Contact (C4C) service to identify people using CMHTs who have already consented to be approached by researchers to take part in research projects.

Once potential participants have been identified, they will be contacted by a researcher who will explain the purpose and nature of the study to them. If they are interested in taking part, a researcher will meet with them and give them the patient information sheet to read and answer any questions. If possible these meetings will be face-to-face, if not they will be by telephone or on Microsoft Teams. If they are still interested in taking part, they will complete a written consent form. If the meeting is not face-to-face, consent forms can either be posted to the participant or they can complete an online consent form. A baseline assessment will then be done which will also be either face-to-face or remotely. After this assessment, they will be allocated to either the intervention or control group (see “Randomisation” section for details below).

Participants in the intervention group will then follow the intervention as outlined above. Participants in the control group will receive a copy of a workbook on managing physical health and a ‘Participant Physical Health Journal’. Both groups will be asked to complete follow-up assessments at 9 months and 15 months after baseline. All participants will be reimbursed £10 for each assessment, £30 in total.

If any participants lose mental capacity during the trial, they will be withdrawn from the study.

### Control group

Participants in the control group will receive treatment as usual from their CMHT regarding their physical health, and we will record what support participants are given for their physical health as there is no agreed practice for this. They will receive a copy of a workbook on managing physical health which was developed by SLaM and includes sections on how people can look after their physical health and well-being at home and details of physical health tests that can be given when people are inpatients, and a copy of the ‘Participant Physical Health Journal’ which is given to participants in the intervention group.

### Randomisation

The randomisation system randomly sequences the order of the participants and enters them into the study stratifying by borough. A random number generator will be used to assign participants to the intervention or the control arm. A statistician independent of the study will do this and put the randomised numbers into sealed envelopes and give these to the research team. This will be put into a list that will be concealed from the research team. When the research team do the initial assessment with a participant, the envelope will be opened to reveal which arm of the study the participant is in after the initial assessment has been completed.

### Sample size

As this is a pilot RCT, we will not undertake a power calculation to calculate the sample size as would be done in a full RCT. We wish to have a sample size of 100 participants—50 in the intervention group and 50 in the control group. As we know there will be participants who drop out of the study, we will recruit 120 participants—60 in the intervention group and 60 in the control group to account for this drop out. We are basing this dropout rate partly on a pilot study undertaken with this population evaluating an intervention to support people to be more physically active [20]. This sample size is used due to the resources of the study.

## Data collection

### Aim 1: Clinical effectiveness of the intervention

#### Primary outcome

Our primary outcome is physical health-related quality of life which we will measure using the EQ-5D-5L [21] at baseline, at the end of the intervention (nine months after baseline) and 6 months after the end of the intervention.

#### Secondary outcomes

The following outcomes will be measured at baseline, and at the end of the intervention (9 months after baseline) and 6 after the end of the intervention.

1. Self-management using the 10-item Patient Activation Measure [22].
2. Mental Health related Quality of Life using the 10-item Recovering Quality of Life (ReQoL) measure [23].
3. Treatment burden using the 10-item Multimorbidity Treatment Burden Questionnaire [24].
4. Loneliness using the 6-item De Jong Gierveld Loneliness Scale [25].
5. We will record participant’s use of physical health services and physical health screenings.
6. We will record demographic information including age, gender, ethnicity, educational level, living arrangements, employment status and relationship status.

### Aim 2: Implementation effectiveness of the intervention

#### Primary outcome

Our primary outcome is acceptability of the intervention measured using the Acceptability of Intervention Measure [17].

#### Feasibility outcomes

1. Feasibility of the intervention measured using the Feasibility of Intervention Measure [17] and qualitative interviews with participants and Health Champions.
2. Appropriateness of the intervention to both participants and Health Champions measured using the Intervention Appropriateness Measure [17] and qualitative interviews with participants and Health Champions.
3. Fidelity to the intervention which we will measure using a content analysis of Health Champions and participants journals to assess fidelity of the delivery of the intervention and fidelity of receipt of the intervention, supervision records and the number and percentage of meetings between each Health Champion and participant.
4. Barriers and facilitators and unintended consequences will be measured using qualitative interviews with a subset of 30% participants and Health Champions chosen at random after the intervention recording whether the intervention was delivered face to face or remotely plus details from the journals and supervision records. We will also interview all participants who drop out of the intervention to understand their reasons for this.
5. Adoption of the intervention will be measured by the number and percentage of participants that start the intervention, how many sessions they have and how long (in months) they stay in the intervention.
6. Adoption and sustainability will also be assessed using interviews with SLaM managers and commissioners.

### Data analysis for aims 1 and 2

#### Quantitative data

Feasibility studies are not designed to detect a treatment effect. It is recommended that feasibility trials should report primarily descriptive statistics on variables that would inform a larger scale RCT [26, 27]. Our data analysis plan is conducted on these premises.

The first stage of analyses will be a descriptive model of the data to assess completeness of data. Participant-level baseline variables will be described both overall and by randomised groups and borough. Patterns of missing data will be described.

The primary outcome will be analysed using linear mixed models, to model the change in scores in EQ-5D-5L as a measure of quality of life. The linear mixed models will be adjusted for baseline total score of EQ-5D-5L and stratification factors (borough). The model will use a random intercept to account for clustering. A two-level hierarchical model will be employed with all timepoints included as repeated measures in the model (after intervention and 9 months) to improve power and to take into account clustering of the observation at patient and group therapy level within each arm of the intervention. These models use maximum likelihood estimation and thus allow for missing outcome data under the missing at random (MAR) assumption. Secondary outcomes will be assessed with a similar methodology for the primary outcomes, using generalised linear mixed models depending on the type of outcome (normal, binary and count). The EQ-5D-5L and other questionnaires to be used have validated methods of scoring, and the scores will be analysed as described to capture change post-intervention and 9-month follow-up (vs. pre-intervention). As this is a pilot study designed to primary measure the feasibility of the trial (and is not focusing to test effectiveness or powered to do so) and to understand if the intervention does impact on the EQ-5D-5L score within each arm of the intervention, we will use any potential change observed on the EQ-5D-5L score to guide a larger trial.

#### Qualitative data

All interviews will be digitally recorded and transcribed then fully anonymised interview transcripts will be produced. Both inductive and deductive coding will be undertaken so that themes will be determined by the interview topic guides and will also be unprompted by the semi-structured nature of the interviews. Thematic analysis [28] will be used to guide the synthesis of the qualitative findings.

### Aim 3: Health economic evaluation of the intervention and implementation

#### An economic evaluation will be used to answer whether

1. The Health Champion approach warrants scaling-up given available evidence on implementation and intervention costs, evidence-based assumptions regarding the effectiveness of its implementation (e.g. anticipated levels of population ‘reach’) and likely impact of the intervention on quality of life and survival in the short- and longer-term.
2. The extent of uncertainty in how cost-effective it would be to scale-up the Health Champion intervention for people with SMI, and the implications of decision uncertainty for commissioning choices.

The economic evaluation will be undertaken using decision analytic modelling approaches combined with a review and synthesis of current clinical, economic and epidemiological evidence (including that generated as part of the feasibility trial) of relevance to quantifying the consequences and cost-effectiveness of available decision options concerning scale-up. In support of this approach, the feasibility trial will be used as an opportunity to generate additional evidence and information necessary to support economic modelling.

The scores from the EQ-5D-5L will be used to calculate health-related quality of life changes and for estimating QALY outcomes. We will calculate the costs associated with the implementation of the intervention (time of volunteer coordinator and the Health Champions) and time taken to complete the intervention (by service users). We will also evaluate the cost of any changes in wider service user by participants.

### Data management

Each participant will be given an identification number. All information collected will be kept confidential; all identifiable data will be kept in a locked cabinet or in password protected documents and forms with identifiable data will be kept separate from the outcome data. Data quality will be enforced by having range checks, valid values and data double-entry. Any changes to the protocol will be reported to the research ethics committee. The final dataset will be accessed by the principal investigator and the research team. The protocol, the anonymised participant level dataset and any statistical codes used will be made available on request.

## Discussion

The Health Champions for people with SMI is a novel and complex intervention, which is based on volunteers supporting people who use mental health services with their physical health. This study will allow us to understand the feasibility of deploying the intervention itself and also its evaluation suite of measures—which capture clinical, implementation and health economic elements.

Upon completion of the study, we will have a detailed overview of whether delivery and planned evaluation approach are feasible to deliver at the scale that will be required of a definitive trial; and also whether participants are able to set and meet goals and feel more in control of their health, as per the original conceptualisation of the Health Champions intervention. Importantly, the design of the study as a hybrid type II trial will allow us to develop our understanding of how the intervention is implemented and the challenges in doing this and also whether the intervention can be sustained. Lastly, the study will allow us to examine the cost implications of the intervention, regarding both the cost of the intervention and its implementation, including how the intervention may impact on costs elsewhere in the healthcare system.

Improving the physical health of people with SMI is a high priority in both policy and practice, having been somewhat neglected in the past. There are currently few evidence-based interventions to support people with SMI with their physical health. If this trial is successfully implemented, it will pave the way for scaled deployment and evaluation of the Health Champions intervention—and potentially inspire further interventions of similar nature for people with SMI to improve their quality of life.

## Data Availability

The datasets used and/or analysed during the current study are available from the corresponding author on reasonable request.

## Abbreviations

SMI: Serious mental illness
RCT: Randomised controlled trial
CMHT: Community Mental Health Team
SLaM: South London and Maudsley NHS Foundation Trust
IMPHS: Integrated Mental and Physical Health Systems
KHP: King’s Health Partners
ToC: Theory of Change
DBS: Disclosure and Barring Service
VSO: Voluntary Sector Organisation
MAR: Missing at random
